# Rapid and Label-Free Isolation of Tumour Cells from the Urine of Patients with Localised Prostate Cancer Using Inertial Microfluidics

**DOI:** 10.3390/cancers12010081

**Published:** 2019-12-29

**Authors:** Alexey S. Rzhevskiy, Sajad Razavi Bazaz, Lin Ding, Alina Kapitannikova, Nima Sayyadi, Douglas Campbell, Bradley Walsh, David Gillatt, Majid Ebrahimi Warkiani, Andrei V. Zvyagin

**Affiliations:** 1 ARC Centre of Excellence for Nanoscale BioPhotonics, MQ Photonics, Macquarie University, 2109 Sydney, Australia; 2 Institute of Molecular Medicine, Sechenov First Moscow State Medical University, 119991 Moscow, Russia; 3 Department of Clinical Medicine, Faculty of Medicine and Health Sciences, Macquarie University, 2109 Sydney, Australia; 4 School of Biomedical Engineering, University of Technology Sydney, 2007 Sydney, Australia; 5 Department of Molecular Sciences, Macquarie University, 2109 Sydney, Australia; 6 Minomic International Ltd., 2113 Sydney, Australia

**Keywords:** prostate cancer, inertial microfluidics, cell separation, tumour cells, glycoprotein

## Abstract

During the last decade, isolation of circulating tumour cells via blood liquid biopsy of prostate cancer (PCa) has attracted significant attention as an alternative, or substitute, to conventional diagnostic tests. However, it was previously determined that localised forms of PCa shed a small number of cancer cells into the bloodstream, and a large volume of blood is required just for a single test, which is impractical. To address this issue, urine has been used as an alternative to blood for liquid biopsy as a truly non-invasive, patient-friendly test. To this end, we developed a spiral microfluidic chip capable of isolating PCa cells from the urine of PCa patients. Potential clinical utility of the chip was demonstrated using anti-Glypican-1 (GPC-1) antibody as a model of the primary antibody in immunofluorescent assay for identification and detection of the collected tumour cells. The microchannel device was first evaluated using DU-145 cells in a diluted Dulbecco’s phosphate-buffered saline sample, where it demonstrated >85 (±6) % efficiency. The microchannel proved to be functional in at least 79% of cases for capturing GPC1+ putative tumour cells from the urine of patients with localised PCa. More importantly, a correlation was found between the amount of the captured GPC1+ cells and crucial diagnostic and prognostic parameter of localised PCa—Gleason score. Thus, the technique demonstrated promise for further assessment of its diagnostic value in PCa detection, diagnosis, and prognosis.

## 1. Introduction

According to GLOBOCAN 2018, prostate cancer (PCa) is the second most frequent cancer as well as the second leading cause of the cancer deaths in males worldwide [1]. Statistical reports show that only a minor decrease in cancer-related mortality has been achieved during the past decade. This was primarily due to a lack of efficient techniques for detection and prognosis of PCa, as well as monitoring the treatment outcomes [2].

Currently, the gold standard for PCa detection is a costly multistage process, which conventionally includes the prostate-specific antigen (PSA) blood test and subsequent tissue biopsy. However, these diagnostic techniques have significant limitations. For instance, the PSA test is sensitive to diverse disorders, including benign prostatic hyperplasia and prostatitis; therefore, its specificity is relatively low [3]. On the other hand, the tissue biopsy is a highly specific yet invasive examination with possible adverse side effects as local bleeding and infectious complications including drug resistant bacterial strains. These shortcomings call for development of cost-effective and reliable alternatives to the existing diagnostic tests for PCa, which would first of all reduce the number of unnecessary tissue biopsies. Liquid biopsy via isolation of circulating tumour cells (CTCs) is a promising diagnostic tool capable of supplementing state-of-the-art PCa diagnostics [4]. However, standard liquid biopsy of the blood samples from PCa patients suffers from low CTC yield, especially in the case of the localised forms of cancer, implying a requirement for providing a large volume of blood for PCa detection. This requirement makes the feasibility of the standard liquid biopsy questionable. Urine represents an obvious and natural choice of the biological fluid to yield diagnostically relevant amounts of cancer cells from patients with diverse urologic cancers including PCa [5,6]. Since a prostate gland is anatomically connected to the lower part of the urinary tract in males, diverse cells can be shed from the gland into the urinary tract, including tumour cells, which can be concomitantly washed out in the process of urination. The major advantages of the tumour cell isolation via liquid biopsy of the urine in comparison with the isolation of CTCs from the blood, as a truly non-invasive method, is a lack of limitation in the sample volume. Compared with blood, urine can be readily collected, stored, and transported. The urine collection process has enviable patient compliance and does not require a skilled medical professional. 

Recently, isolation of tumour cells from biological samples via microfluidic technology has gained significant attention. Among the existing microfluidic modalities, inertial microfluidics based on a spiral microchannel device configuration has experienced a massive growth as exemplified by the separation of CTCs and circulating fetal trophoblasts [7,8,9,10], identification of bacterial spoilage from beers [11], blood fractionation [12], enrichment of circulating head and neck tumour cells [13], and separation of microalgae [14]. The technique is attractive for its cost-effectiveness and high throughput. The technology exploits inertial lift and Dean drag forces exerted on cells and particles flowing through the spiral microchannel. The interplay of these two forces causes lateral migration of cells and their focussing at equilibrium positions. As the amount of these two forces is directly related to the cell size, the cell eventually equilibrates at a lateral position, which precisely corresponds to its diameter. As a result, the larger cells (or particles, or both) tend to focus at regions near the inner wall, whereas the smaller cells (or particles, or both) tend to move away from it. 

In this communication, we report on the development of a microfluidic chip capable of collecting PCa tumour cells from the urine using the principles of cell sorting employing inertial microfluidics. Isolated tumour cells were assayed via immunocytochemistry with monoclonal mouse anti-glypican-1 (anti-GPC-1) [15] antibody MIL-38 (Minomic International Ltd., Sydney, NSW, Australia) previously reported to be highly specific and sensitive in the detection of PCa [16]. MIL-38 was used as a model primary antibody. The GPC-1 is a heparan sulphate proteoglycan which is found attached to the cancer cell surface and which has recently received significant attention as a biomarker for PCa, especially in terms of evaluating its aggressiveness and growth [17]. Furthermore, to showcase the versatility of the developed microchannel in terms of diagnosis and prognosis of localised PCa, correlations between the number of isolated tumour cells and conventional diagnostic parameters of PCa such as the level of prostate-specific antigen (PSA) in blood and Gleason score (GS) were analysed.

## 2. Methods

### 2.1. Device Design and Fabrication

A spiral microchannel device for the isolation of PCa cells from urine samples was designed, fabricated, and tested. This device represented an adaptation of the microfluidic technology previously developed for the isolation of CTCs from peripheral blood [9,18]. Briefly, polydimethylsiloxane (PDMS) pre-polymer with the curing agent (Sylgard 184, DowsilCorp., Midland, MI, USA) were first mixed at the ratio of 10:1 and then was degassed in a vacuum chamber. This mixture was poured onto an aluminium mold with subsequent baking in a laboratory oven for 2 hours at 70 °C. The mold can be fabricated via a milling process [19] or 3D printing technology [20]; in this study, we used a milling process. Furthermore, the cured PDMS was peeled from the mold, and holes with 1.5 mm diameter were pierced with a Uni-Core™ Puncher (Sigma-Aldrich Co. LLC., Singapore) at the sites designated for the inlet and outlets of the chip. Eventually, the PDMS microchannel was irreversibly bonded to another layer of PDMS using an oxygen plasma machine (Harrick Plasma, Ithaca, NY, USA). 

### 2.2. Experimental Setup and Procedure 

The efficiency of the spiral microfluidic chip for capturing PCa tumour cells was evaluated by spiking a predetermined number of DU-145 cells (ATCC HTB-81) (approximately 1000) into 50 mL of Dulbecco’s phosphate-buffered saline (DPBS). The solution was injected into the chip using a peristaltic pump (Baoding Shenchen Precision Pump Co., Ltd, Baoding, China) at a 1.7-mL/min flow rate. When the volume of DPBS in the sample tube decreased to 1 mL, enriched cells were deposited onto an adhesive glass slide (ThermoFisher Scientific, Scoresby, VIC, Australia) using a Thermo Scientific™ Cytospin™ 4 Cytocentrifuge (ThermoFisher Scientific, Scoresby, VIC, Australia) and then air-dried. To determine if there were cells in the waste outlet, DPBS in the waste tube was centrifuged, and cell pellets were deposited onto 4 adhesive glass slides with Cytospin™ 4 Cytocentrifuge for further quantitation. Deposited cells on slides were fixed with ice-cold acetone for 3 min at –20 °C and mounted with a Fluoroshield medium containing 4′,6-diamidino-2-phenylindole (DAPI) Prolong Gold Antifade Reagent(ThermoFisher Scientific, sScoresby, VIC, Australia) covered with a coverslip and counted using a fluorescence microscope (Zeiss Axio Imager Z2 Upright Microscope, North Ryde, NSW, Australia).

### 2.3. Ethics Statement and Clinical Sample Preparation

**Figure 1:**
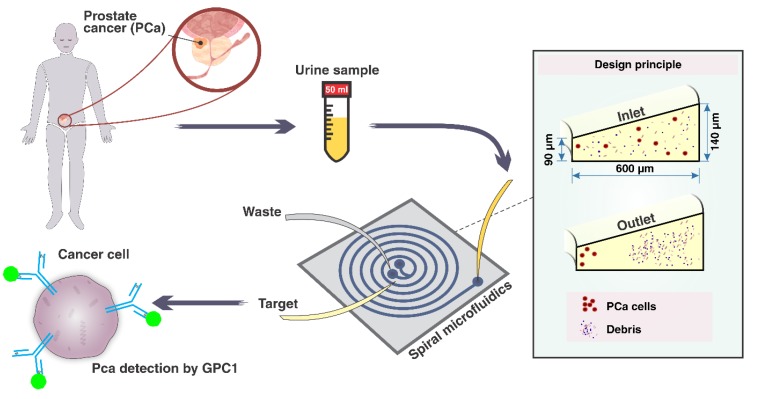
A schematic representation of the workflow for PCa cell detection from the urine sample employing a spiral microfluidic chip. First, PCa cells shed from the prostate gland into the urethra are collected into a container in the process of urination. Then, the collected PCa cells present in urine are isolated via processing through the spiral microchannel. Finally, the collected cells are labelled with fluorescent antibodies, i.e., anti-GPC-1 and immunoassayed under a microscope.

A part of this study that includes collection and analysis of clinical samples from patients with localised PCa was conducted under an ethical approval provided by Macquarie University Human Research Ethics Committee (HREC No: 5201500707), Australia. All patients and healthy volunteers provided written informed consent for collection of the samples and provision of their clinical data. The samples were collected and analysed in a non-blinded manner, 14 midstream urine samples from healthy volunteers and fourteen midstream urine samples from patients with localised PCa were acquired. The midstream specimen of urine was chosen for analysis to avoid possible channel occlusion with debris which may present in the first pass specimen. The volume of urine collected from patients and healthy volunteers varied from 30 to 100 mL. Prior to sample processing, the amount of cells per mL of urine was identified with an automated cell counter (TC20™ Automated Cell Counter, Bio-Rad, Gladesville, NSW, Australia). After the sample collection, samples were processed through the spiral microchannel on the same day with the peristaltic pump at the optimum flow rate of 1.7 mL/min. During the sample processing, once the volume reduced to approximately 5 mL, 5 mL of DPBS was added, and the processing was continued until the volume of solution decreased to approximately 1 mL. Afterwards, cells from the solution were sedimented with Cytospin™ 4 Cytocentrifuge onto four adhesive glass slides. The glass slides were then air-dried and fixed with ice-cold acetone for 3 min at –20 °C and processed either immediately or stored at 4 °C in a dry chamber and analysed within a week. The schematic representation of the sample processing is illustrated in Figure 1. 

### 2.4. Immunofluorescence Staining

All incubations were performed in a humidified chamber. Cells were fixed on the surface of dry glass slides, rehydrated in DPBS for 3 min, followed by blocking with 1% Bovine Serum Albumin (BSA) solution in DPBS for 1 h at room temperature. Cells were then incubated for 2 h at room temperature with an MIL-38 anti-GPC-1 mouse primary antibody diluted in blocking solution (1% BSA) at 10 μg/mL. At this step, one of the 4 slides was incubated with the blocking agent instead of the secondary antibody and was used as a secondary antibody alone control to monitor the non-specific binding of second antibody. Then, the glass slides were washed with DPBS 3 times for 5 min and air-dried. Then, cells were incubated for 1 h with goat-anti-mouse Alexa Fluor 488 (Abcam, Australia) diluted in the blocking solution at 4 μg/mL and washed with DPBS 3 times for 5 min after the incubation. Finally, cells were mounted with a Fluoroshield medium containing DAPI, covered with a coverslip, and observed under a fluorescence microscope.

### 2.5. Cell Enumeration and Data Analysis

To identify a quantitative gold standard for enumeration of GPC-1^+^ putative tumour cells collected from the urine samples, approximately DU-145 cells were added to a healthy urine sample, processed with the chip, immunostained as described above, captured under the fluorescence microscope, and the intensity of fluorescence signal from the cells was measured with ImageJ software. Then, a mean (±SD) for the signal intensity was calculated, and the intensity at the value equal to mean–SD was chosen as the threshold. Above this threshold, cells from the urine samples of PCa patients or healthy volunteers were registered as putative tumour cells and counted. Thus, in the case of cells isolated from the urine sample of PCa patients, during observation under the microscope, images of suspicious cells were captured, and the cells were analysed with ImageJ software for signal intensity. GPC-1^+^ cells whose signal intensity was higher than the mean–SD (determined using DU-145 cells) were registered as putative tumour cells and counted. Finally, to evaluate potential diagnostic and prognostic value of the technique, correlations between the amount of GPC-1^+^ cells collected from the patients and the levels of conventional PCa markers such as the blood PSA level and GS were measured. To further evaluate the applicability of the developed microfluidic device, correlations between the amount of collected GPC-1^+^ cells, the volume of the urine sample and total amount of cells in urine sample (mL^−1^) were measured. The correlations were calculated using Microsoft Excel 2016 software (Microsoft, Redmond, WA, USA).

## 3. Results

### 3.1. Spiral Microfluidic Device 

In this study, we report a novel, efficient, and non-invasive method for isolation of tumour cells from urine samples. This is the first time, to the best of our knowledge, that detection of PCa cells using the urine samples based on inertial microfluidics in a spiral channel has been reported. 

Prostate cancer cells experience two major inertial lift and Dean drag forces inside the spiral channel. These forces can be calculated by Equations (1) and (2):

(1) $$F_{L}=\rho {\left(\frac{U_{max}}{D_{h}}\right)}^{2}C_{L}a^{4},$$

(2) $$F_{d}=5.4\times 10^{-4}\pi \mu De^{1.63}a\;,$$

where $U_{max}$ indicates the maximum fluid velocity, ρ is the fluid density, μ denotes the dynamic viscosity of the fluid, $C_{L}$ is the lift force coefficient, $D_{h}$ represents the channel hydraulic diameter, a is the particle diameter, and De is Dean number. Considering these equations, a particle (or cell) of the diameter a experiences $F_{L}$ and $F_{d}$ different uniquely determined by the a [11]. As schematically shown in Figure 1, the larger cells (prostate cancer cells) are focussed at the inner wall (where its height is purpose-designed to be smaller than that of the outer wall), whereas the smaller cells and debris drift to the outer wall.

**Figure 2:**
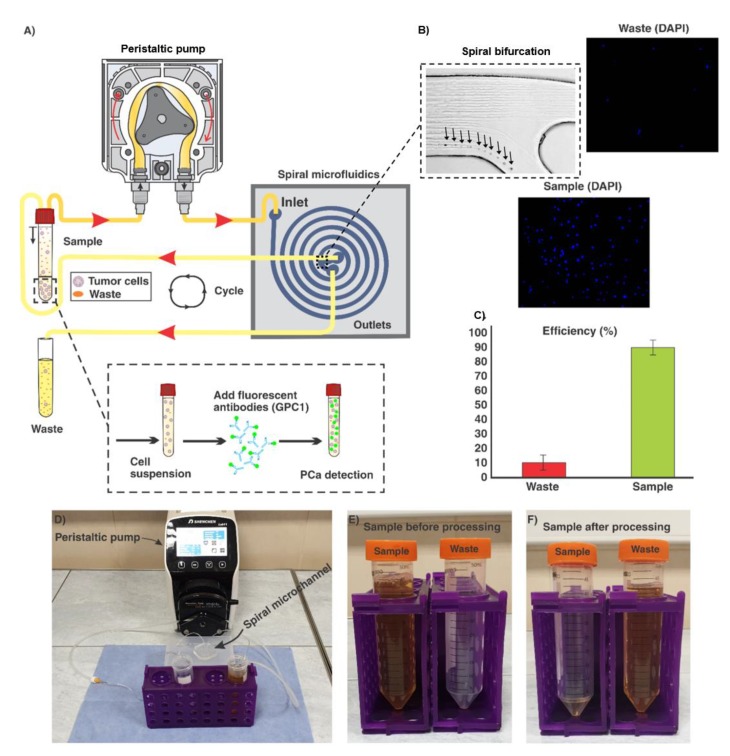
**(A**) schematic representation of the processing of a urine sample containing PCa cells through the spiral microfluidic chip. Samples are introduced via a peristaltic pump and then recycled until 1 mL of the urine sample remains in the sample tube. Then, PCa assaying is implemented by the use of fluorescent antibodies; (**B**) illustration of the bifurcation of the spiral, target (sample), and waste outlets. The results show that most of cells were collected through the target outlet. The flow rate of 1.7 mL/min was selected as the optimum flow rate for the separation efficiency of PCa cells; (**C**) 85 (±6) % of cells were collected through the sample outlet of the chip; (**D**) experimental setup used in this study; (**E**) state of urine sample before processing, and (**F**) after processing, when about 1 mL of sample remains in the tube. The remaining 1-mL contains most of PCa cells and is subsequently analysed as described.

Generally, the ratio of particle diameter to the height of channel, as well as the hydraulic diameter of microchannel play a critical role in the particle focussing. The channel cross-section designed in this study was carefully analysed to focus PCa cells (~15–20 µm) featuring a trapezoidal cross-section with the base of 600 µm, inner wall of 90 µm, and outer wall of 140 µm. The bifurcation at the outlet of the channel was placed at a location 350 µm to the inner wall. Upon the optimisation of the channel flow rate, DU-145 cells in DPBS solution were introduced from the inlet via a peristaltic pump. The results demonstrated that the flow rate of 1.7 mL/min was optimal where 85 (±6) % of cells were collected through the target outlet (Figure 2).

### 3.2. Collection of GPC-1^+^ cells from Urine

**Figure 3:**
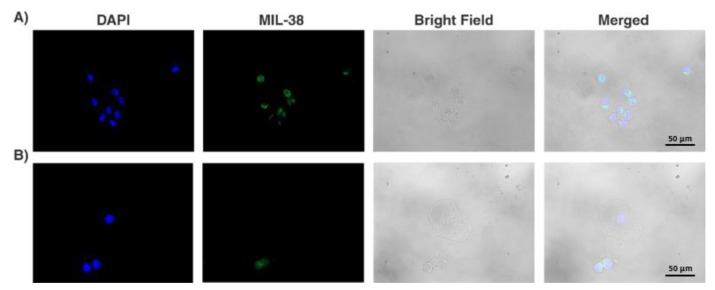
Numerous glypican-1+ (GPC-1^+^) cells (**A**), two GPC-1^+^ cells and one GPC-1^−^ squamous epithelial cell (**B**) isolated from the samples of PCa patients. The GPC-1^+^ putative tumour cells typically featured a high ratio of the nucleus to cytoplasm size and were prone to grouping or clustering.

Midstream urine samples in volumes ranging from 30 to 100 mL were collected from 14 patients with localised PCa and processed using our spiral microfluidic chip. GPC-1^+^ cells exhibiting the fluorescence signal >1781 arbitrary units, according to the mean (2069)-SD (288) arbitrary units measured from DU-145 cells with ImageJ were registered as putative tumour cells. Mostly, these cells exhibited round nuclei and a high ratio of nuclear to cytoplasmic size. We note that these cells were located in groups or clusters (Figure 3A,B). Putative tumour cells were detected in 12 out of 14 patients (86%). The total amount of detected cells (n) varied from 4 to 194 among the samples and patients, with the median value of 22. In the case of healthy volunteers, 11 samples out of 14 (79%) were negative in terms of GPC-1^+^ cells. It is worthy to note that the urine samples of the 3 healthy volunteers registered as GPC-1^+^ positive, contained only <8 cells. Thus, in case of PCa patients, only those patients whose urine samples contained n > 8 were considered positive with confidence. Accordingly, the number of such positive patients was 11 out of 14 (79%) (Table 1). 

Low negative correlation between the amount of GPC-1^+^ cells and the volume of urine sample (*r* = −0.18, where *r* is correlation coefficient) and negligible positive correlation between the amount of GPC-1^+^ cells and the total number of cells per mL of the initial urine sample (*r* = 0.02) were identified. At the same time, moderate positive correlation between the volume of the urine sample and the total number of cells (mL^−1^) (*r* = 0.32) was identified. Among all 28 samples analysed, 1 (4%) sample was discarded due to the high concentration of non-cellular elements (i.e., urine crystals).

### 3.3. Analysis of Potential Diagnostic Applicability of the Method

**Figure 4:**
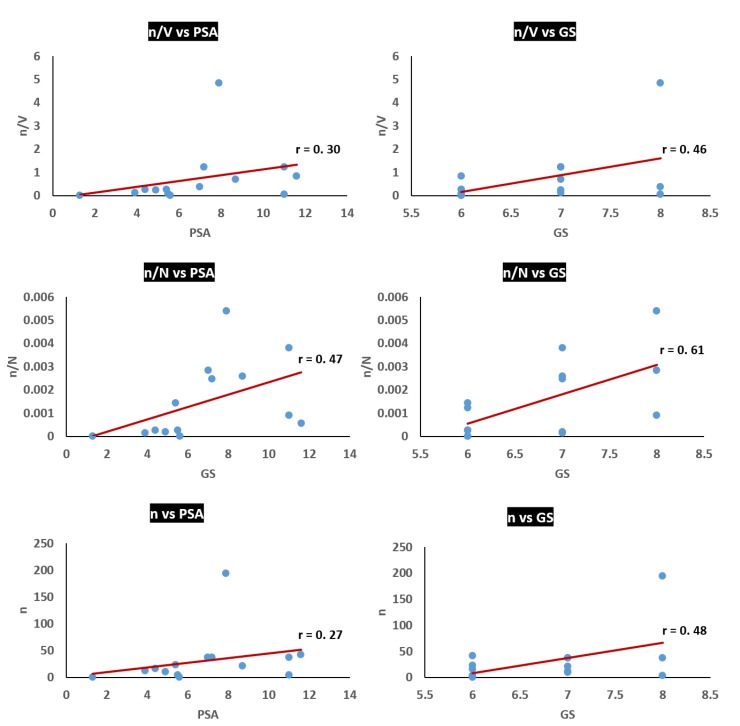
Correlations between the amount of GPC1+ cells (n), n/V (V, volume of urine sample), n/N (N, total number of cells in urine sample) and conventional clinicopathological parameters of the PCa–prostate specific antigen (PSA) level and Gleason score (GS). The lowest correlation was identified between n and PSA level, and the highest correlation was identified between n/N and GS.

To evaluate the flexibility of the method proposed in this study for early detection and diagnosis of PCa, correlations between the amount of isolated putative tumour cells and conventional diagnostic parameters of PCa were analysed. Thus, correlations between the amount of GPC-1^+^ cells (n) and PSA level, or total GS were recognised (Figure 4). Furthermore, correlations between the ratio of n/V (where V is volume of urine sample), or the ratio n/N (where N is a total number of cells in urine sample), and PSA level, or GS, were also identified. As a result, low positive correlations were identified between n and PSA level (*r* = 0.27) as well as n/V and PSA level (*r* = 0.30), moderately positive correlations between *n* and GS (*r* = 0.48), and between n/V and GS (*r* = 0.46), and moderately positive correlations between n/N and PSA level (*r* = 0.47) and GS (*r* = 0.61).

## 4. Discussion

PSA screening is the most common method of early detection of PCa. A prostate biopsy remains the only definitive diagnostic test for the presence of PCa. However, the high prevalence of false positive PSA tests gives rise to a large number of un-necessary prostate biopsies. There remains a high clinical need for tests better able to guide a decision to proceed to prostate biopsy. This provides an opportunity to choose the right time for surgical intervention in the case of the parameter increments [21]. However, the specificity of PSA screening is not high and a GS is obtained via an invasive tissue biopsy causing significant discomfort to a patient. In recent years, early diagnosis and prognosis of PCa by means of CTC isolation via liquid biopsy of blood has been broadly investigated. However, progress has been minimal and the technique is not efficient due to the low number of CTCs in a standard sample of 7.5 mL of peripheral blood [22]. As an alternative, in this study, we developed and investigated a high-throughput microfluidic chip for enrichment of PCa tumour cells derived from the urine.

In the pilot enrichment of DU-145 cells from DPBS, the chip demonstrated 85 (±6)% efficiency in capturing cells at the optimum flow rate i.e., 1.7 mL/min. For urine sample analysis, 12 out of 14 PCa patient samples (86%) were positive, and 11 out of 14 patients (79%) were positive with confidence in terms of cells with the high level of GPC-1^+^ expression—the cells which were registered as putative tumour cells in the current study. At the same time, 11 out of 14 healthy volunteers (78%) were identified as PCa-negative in terms of GPC-1^+^ cells. Such results corresponded to the specificity and sensitivity of anti-GPC1 primary antibody MIL-38 used as a model primary antibody for the detection of putative tumour cells [16]. A median amount of GPC-1^+^ cells of 22 units, captured from patient samples, identifies urine as a preferable medium for liquid biopsy of localised PCa. Furthermore, low negative correlation (*r* = −0.18) identified between the amount of captured GPC-1^+^ cells (n) and the volume of the urine (V) suggested that the major amount of n were released in the first stream of the urine—a reasonable assumption considering the anatomical connection of the prostate gland to urethra [23]. This assumption is corroborated by moderately positive correlation between the total number of cells in urine samples (N) and V (*r* = 0.32) alongside a positive correlation between N and n (*r* = 0.02). This seems to indicate that other cells normally present in urine are gradually released during urination. 

The low positive correlation between n/V and PSA level (*r* = 0.30) suggests that larger volumes of urine do not contain proportionally greater n, and the first stream of urine is sufficient to collect the majority of PCa cells. Therefore, it can be concluded that ≤30 mL of the voided urine is likely to be optimal for rapid isolation of tumour cells using our spiral microfluidic chip. The low positive correlation between n and PSA level (*r* = 0.27) can be explained by the relatively low specificity of the PSA level increment as a marker for the PCa progression and aggressiveness [24]. However, the moderately high correlation between n/N and PSA level (r = 0.47) is noteworthy. The highest positive correlation in this study was achieved between n/N and GS (*r* = 0.61) and can be explained by the high specificity of GS as a marker of the progression and aggressiveness of localised PCa [25]. This assumption is corroborated by the moderate correlation between n and GS (*r* = 0.48) and between n/V and GS (*r* = 0.46).

The reported technique lends itself to some straightforward improvements. Fine-tuning of the chip by including an additional outlet to remove large waste elements will enable handling of a greater variety of urine samples, including samples containing a large amount of urine crystals, which we had to discard. The use of more specific and sensitive PCa antibodies or their combination will improve the immunocytochemistry assaying and increase the sensitivity and specificity of our technique.

## 5. Conclusions

For the first time, to the best of our knowledge, we introduce here a spiral microfluidic chip capable of rapid and label-free isolation of tumour cells from urine. The spiral microchannel used in this study had a trapezoidal cross-section with a width of 600 µm, an inner wall of 90 µm, and outer wall of 140 µm. The microchannel was first tested using a spiked cancer cell line, proving its high efficiency: separation of ca 86% of cancer cells at the optimum flow rate of 1.7 mL/min. Secondly, ≥79% of the analysed clinical samples from urine of patients with localised PCa were positive for GPC-1^+^ putative tumour cells. Thirdly, moderate correlations were observed between the ratio of GPC-1^+^ cells (n) to the total number of isolated cells (N) and PSA; n and GS; and n/V (V, urine volume) and GS. These results demonstrate promise of the spiral inertial microfluidic technique in terms of diagnosis and prognosis of localised PCa by liquid biopsy of urine, paving the way for inexpensive rapid, non-invasive diagnosis, as well as screening and monitoring therapeutic outcomes of PCa and other urology cancers.

## Figures and Tables

**Table 1:** The number of glypican-1^+^ (GPC-1)^+^ cells detected in the urine samples and relevant clinical diagnostic test results. The number of cells with high GPC-1^+^ expression (n ) isolated from the patients’ and urine samples varied from 4 to 194 units with the median value of 22. n was <8 in the urine samples of the healthy volunteers, i.e., 11 (79%) out of 14 healthy volunteers were registered PCa-negative in terms of the n -number. The number of patients positive with confidence for GPC-1^+^ cells (n > 8) was 11 out of 14 (79%).

Patient Number	*n*	Urine Sample Volume (mL)	N	Blood PSA level (ng/mL)	Total GS
1	23	90	1.6 × 10^4^	5.4	6
2	21	30	8.1 × 10^3^	8.7	7
3	37	100	1.3 × 10^4^	7	8
4	16	60	6.3 × 10^4^	4.4	6
5	4	60	4.4 × 10^3^	11	8
6	10	40	5.2 × 10^4^	4.9	7
7	37	30	1.5 × 10^4^	7.2	7
8	12	90	8.3 × 10^4^	3.9	7
9	194	40	3.6 × 10^4^	7.9	8
10	42	50	7.4 × 10^4^	11.6	6
11	0	40	1.9 × 10^4^	1.3	6
12	37	30	9.7 × 10^3^	11	7
13	11	50	1.5 × 10^4^	5.5	6
14	0	70	2.7 × 10^3^	5.6	6

*n*—number of GPC1+ cells; N—total number of cells in the analysed sample.
